# Bibliometric Analysis of 100 Most-Cited Articles in Delirium

**DOI:** 10.3389/fpsyt.2022.931632

**Published:** 2022-07-06

**Authors:** Xinxing Fei, Qiu Zeng, Jianxiong Wang, Yaqian Gao, Fangyuan Xu

**Affiliations:** ^1^Department of Psychiatry Chengdu Eighth People's Hospital (Geriatric Hospital of Chengdu Medical College), Chengdu, China; ^2^Department of Rehabilitation Medicine, The Affiliated Hospital of Southwest Medical University, Luzhou, China; ^3^Department of Rehabilitation Medicine, The First Affiliated Hospital of Chengdu Medical College, Chengdu, China

**Keywords:** bibliometric analysis, bibliometrics, delirium, psychiatry, cognitive disorder, top-100

## Abstract

Delirium is a cognitive disorder with complex etiology, which brings a great burden to social health care. Articles with high citation frequency can provide important information about the current research situation in a certain field. Web of Science was used to search the 100 most-cited articles and we extracted key information, such as the authors, countries/regions, institutions, journals, and study types of these articles. CiteSpace was used to visually analyze the keywords. Our bibliometric analysis shows that the attention in this field continues to rise. Authors from the United States published the most articles and Inouye SK is the most influential author in the field. The journals that published these articles have high impact factors. Cohort studies are the main cited articles in this field, and there are a large number of systematic reviews or meta-analyses of cohort studies. Risk factors for delirium, psychometric evaluation, hospital care, and various clinical study design are still the focus of research. In short, we summarized the 100 most-cited articles in the field of delirium to identify the current status and global trends. These results enable researchers to understand the quality and trend of research in the field of delirium and make better use of the classical literature.

## Introduction

Delirium is a non-specific, preventable, and reversible cognitive disorder characterized by acute changes in mental state, inattention, and confusion of thinking (1). The cause of delirium is complicated, which is often caused by various promoting factors on the basis of risk factors, such as advanced age and cardiopulmonary dysfunction (2, 3). The incidence and prevalence rate varies greatly according to the clinical situation and population (4). The occurrence of delirium can not only lead to longer hospital stays and increased medical expenses but also affect the long-term cognitive ability of patients without timely treatment (5, 6). Therefore, early identification of the causes of delirium and correction of risk factors can improve the symptoms of delirium and reduce the burden on society and family (7).

Bibliometric analysis is an important tool to evaluate research performance and identify influential papers in specific fields (8). Articles with high citation frequency can provide important information about the current research situation in a certain field (9). At present, a large number of studies on delirium have been published, including bibliometric analysis in the field of delirium within a certain time frame (10, 11). However, bibliometric analyses of articles with high quality and high citation frequency of delirium have not been reported. Considering the clinical significance of delirium and the importance of highly cited articles, we qualitatively and quantitatively analyzed the 100 most-cited articles in the field of delirium, in order to help researchers to understand the research quality and trends, better use of the classical articles about delirium, and provide a reference for future research in this field.

## Methods

### Database Selection and Search Strategy

Scopus and Web of Science are popular bibliometrics search tools, but Scopus only includes articles published after 1966 (12). Considering that classical articles may be published earlier, we used the Web of Science core collection (index: Science Citation Index Expanded) as the search tool and TI= “delirium” as the search strategy to get the research in the field of delirium on 18 April 2022.

### Data Collection and Analysis

Two researchers sorted the citation frequency of the searched articles from high to low and downloaded the data of top-100 articles for further analysis. Key information, such as author, institution, title, publication year, citation frequency, citation density (total citation frequency divided by the year since publication), journal name, impact factor (IF), and country/region, were extracted.

CiteSpace is a visualization analysis software developed gradually under the background of bibliometrics and data visualization (13). Now it has updated several versions, and the data visualization function is more and more powerful (13). In this study, the CiteSpaceV 5.8.R3 software was used for keywords co-occurrence analysis and cluster analysis (the original data of the 100 most-cited articles has been uploaded to Data Sheet 1). Specific parameters were set according to previous studies as follows (14, 15): “Time Slicing” was set to 1955–2022 (#Years Per Slice = 1); “Term Source” selected “Title,” “Abstract,” “Author Keywords (DE),” and “Keywords Plus (ID)”; “Node Types” selected to “Keywords”; “Links” and “Selection Criteria” used the default option; “Pruning” selected “pathfinder” and “pruning the merged network”; “Visualization” selected “cluster view-static” and “show merged network.”

In addition, some journals changed their names, such as Archives of Internal Medicine to JAMA Internal Medicine. We used the original journal name in the results section and reported the IFs in 2020. Two researchers completed the above process independently. If there was a disagreement between the two researchers, a third researcher would intervene. No patient or public participation in this study, so no ethical approval was required to conduct this study.

## Results

### Publications and Citations

**Figure 1:**
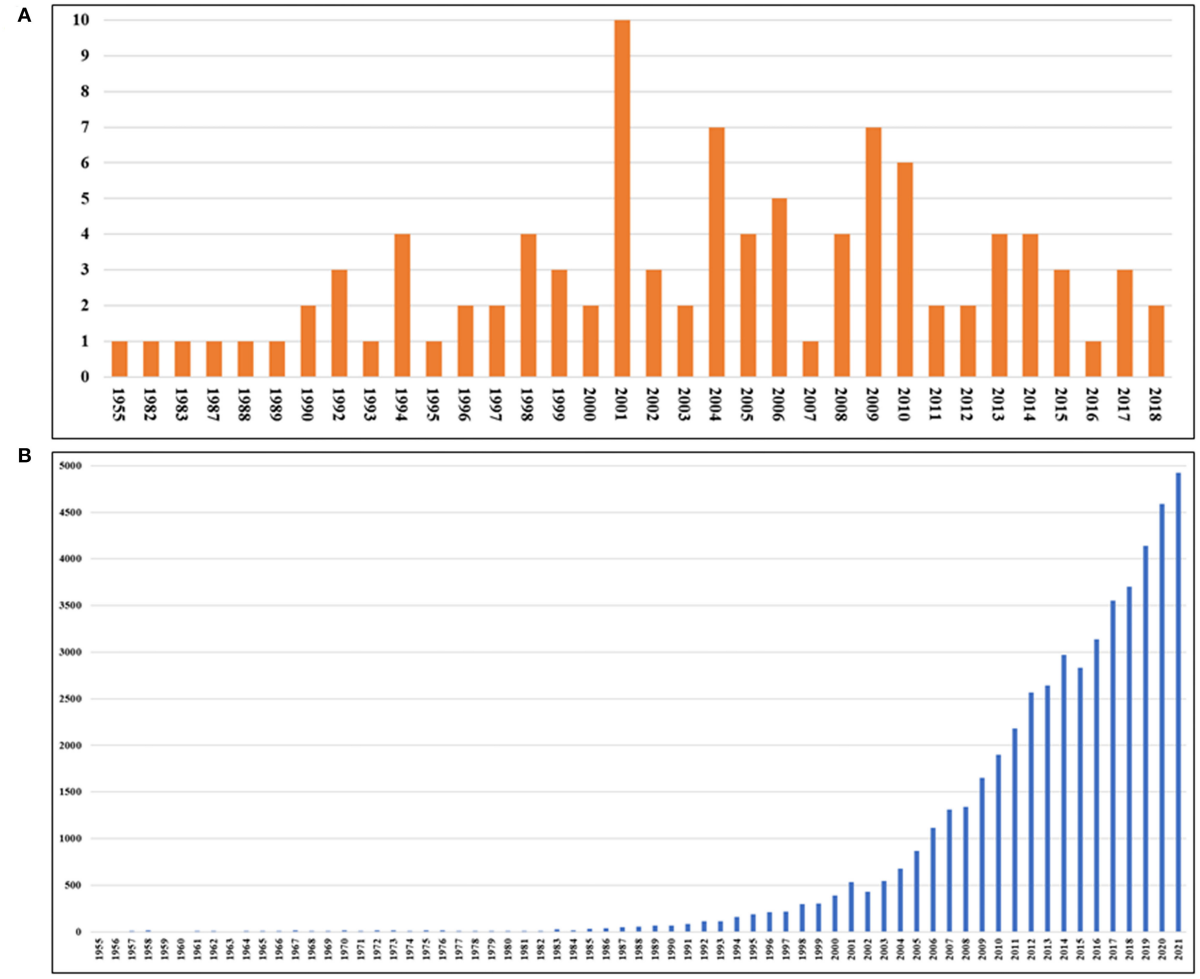
The year of publication **(A)** and total citation frequency each year of all articles **(B)** of the 100 most-cited articles in the field of delirium.

The top-100 articles about delirium searched are shown in Supplementary Table S1. These articles were published between 1955 and 2018, with the largest number of articles published in 2001 (n = 10). The publication of the articles stabilized as a whole after the tipping point of 2001 (Figure 1A). For the citations, the total annual citations of the article increased steadily from 1984 to 2021. Since 2016, there have been more than 3,000 citations per year (Figure 1B).

### Countries/Regions, Institutions, and Authors

**Figure 2:**
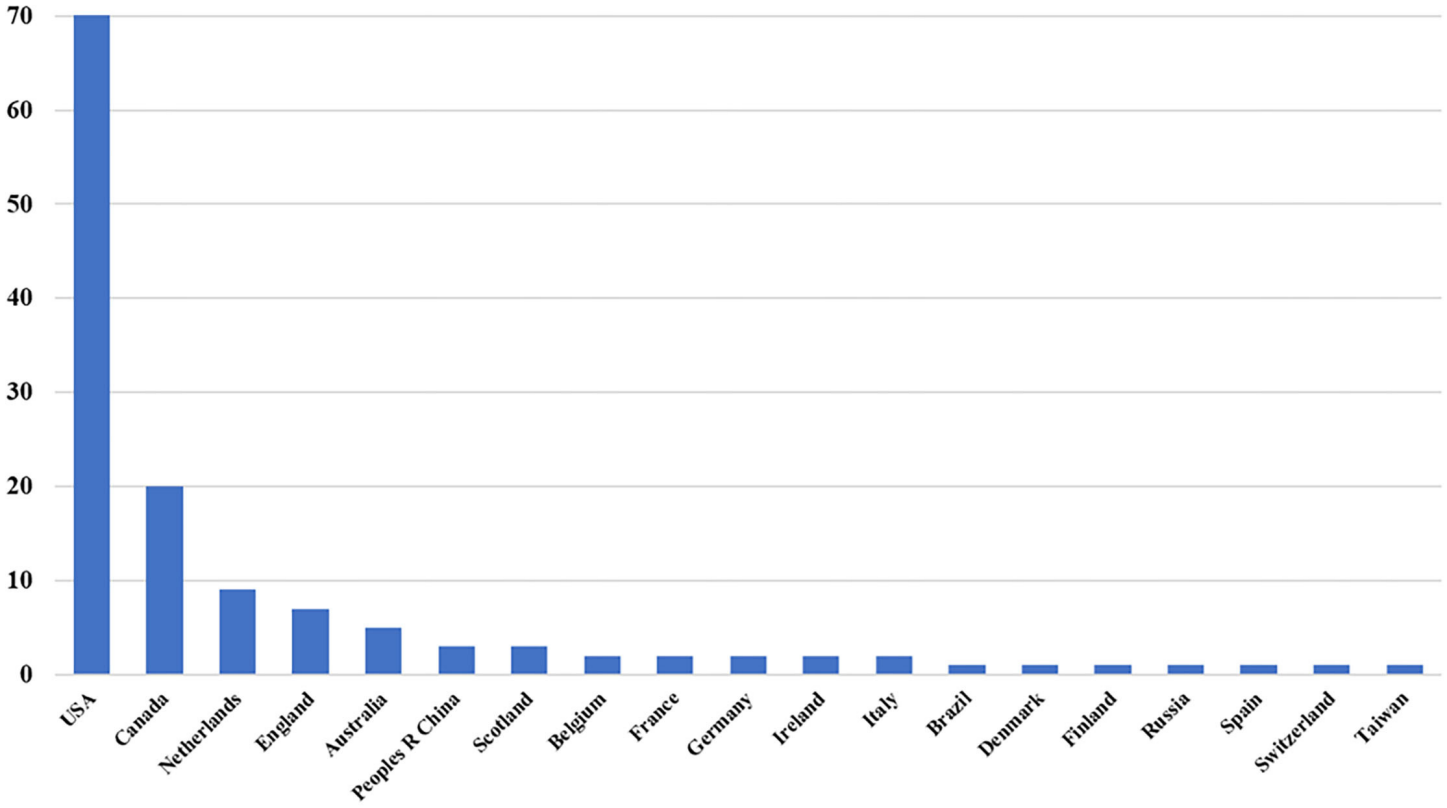
Number of the 100 most-cited articles published by country or region.

The country/region distribution of these papers was analyzed. Authors from the United States published the most papers (*n* = 72), followed by Canada (*n* = 20), Netherlands (*n* = 9), England (*n* = 7), and Australia (*n* = 5). Overall, 19 countries/regions account for the top-100 articles in the field (Figure 2). In addition, we counted the top 10 institutions that published these 100 articles (Table 1). Harvard University published 22 articles, followed by Yale University (*n* = 20), and Vanderbilt University (*n* = 19), which were also institutions with a citation frequency of more than 10,000. Table 2 shows the top 10 authors who have published the most top papers. The author with the most articles was Inouye SK, with a total of 26, and 11 were published as the first author. The total number and average of citations of his articles were also the highest (19,942 and 771.85). Ely EW (*n* = 16), Marcantonio ER (*n* = 13), Bernard GR (*n* = 8), and Pun BT (*n* = 8) ranked second to fifth, respectively.

**Table 1:** Institution with top-10 number of papers included in the top-100 articles.

**Rank**	**Institution**	**Publications**	**Total citations**	**Mean citations**
1	Harvard University	22	10,700	489.18
2	Yale University	20	15,991	803.75
3	Vanderbilt University	19	12,997	689.74
4	US Department of Veterans Affairs	18	9,468	529.44
5	Veterans Health Administration (VHA)	18	9,468	529.44
6	Geriatric Research Education Clinical Center	16	8,976	564.25
7	Brigham and Women's Hospital	13	5,195	401
8	VA Tennessee Valley Healthcare System	13	8,106	627.15
9	Beth Israel Deaconess Medical Center	12	5,391	450.83
10	League Of European Research Universities (LERU)	12	6,006	501.75

**Table 2:** Authors with top-10 number of papers included in the top-100 articles.

**Rank**	**Author**	**Publications**	**As first author**	**Total citations**	**Mean citations**	**Institution**
1	Inouye SK	26	11	19,942	771.85	Yale University
2	Ely EW	16	4	11,707	736.75	Vanderbilt University
3	Marcantonio ER	13	6	5,238	404.92	Hebrew Rehabilitation Center for Aged
4	Bernard GR	8	0	7,497	940.13	Vanderbilt University
5	Pun BT	8	0	3,800	476.63	Vanderbilt University
6	Skrobik Y	7	0	5,084	728.86	University of Montreal
7	Dittus RS	6	0	4,325	722.17	Vanderbilt University
8	Devlin JW	5	3	3,629	727.4	Northeastern University
9	Fong TG	5	2	1,883	377	Harvard University
10	Francis J	5	2	4,442	890.2	University of Pittsburgh

### Journals

The articles were published in 36 journals. The top 10 cited journals, IFs, and Journal Citation Reports (JCR) area are shown in Table 3. According to the JCR zoning, all journals belong to the Q1 zone. Among these journals, the most citation frequency was *Critical Care Medicine* (*n* = 12), followed by *Journal of the American Geriatrics Society* (*n* = 11), and *JAMA-Journal of the American Medical Association* (*n* = 10). And *New England Journal of Medicine* had the highest IF of 91.245. In addition, the IF of 9 journals was more than 5.

**Table 3:** Journals with top-10 number of papers included in the top-100 articles.

**Rank**	**Journal**	**Publications**	**IF_**2020**_**	**JCR**
1	Critical Care Medicine	12	7.598	Q1
2	Journal of the American Geriatrics Society	11	5.562	Q1
3	JAMA-Journal of the American Medical Association	10	56.272	Q1
4	Archives of Internal Medicine (JAMA Internal Medicine)	7	21.873	Q1
5	New England Journal of Medicine	6	91.245	Q1
6	Intensive Care Medicine	5	17.44	Q1
7	Age and Aging	4	10.668	Q1
8	American Journal of Medicine	3	4.965	Q1
9	Anesthesia and Analgesia	3	5.108	Q1
10	Critical Care	3	9.097	Q1

### Study Types

**Figure 3:**
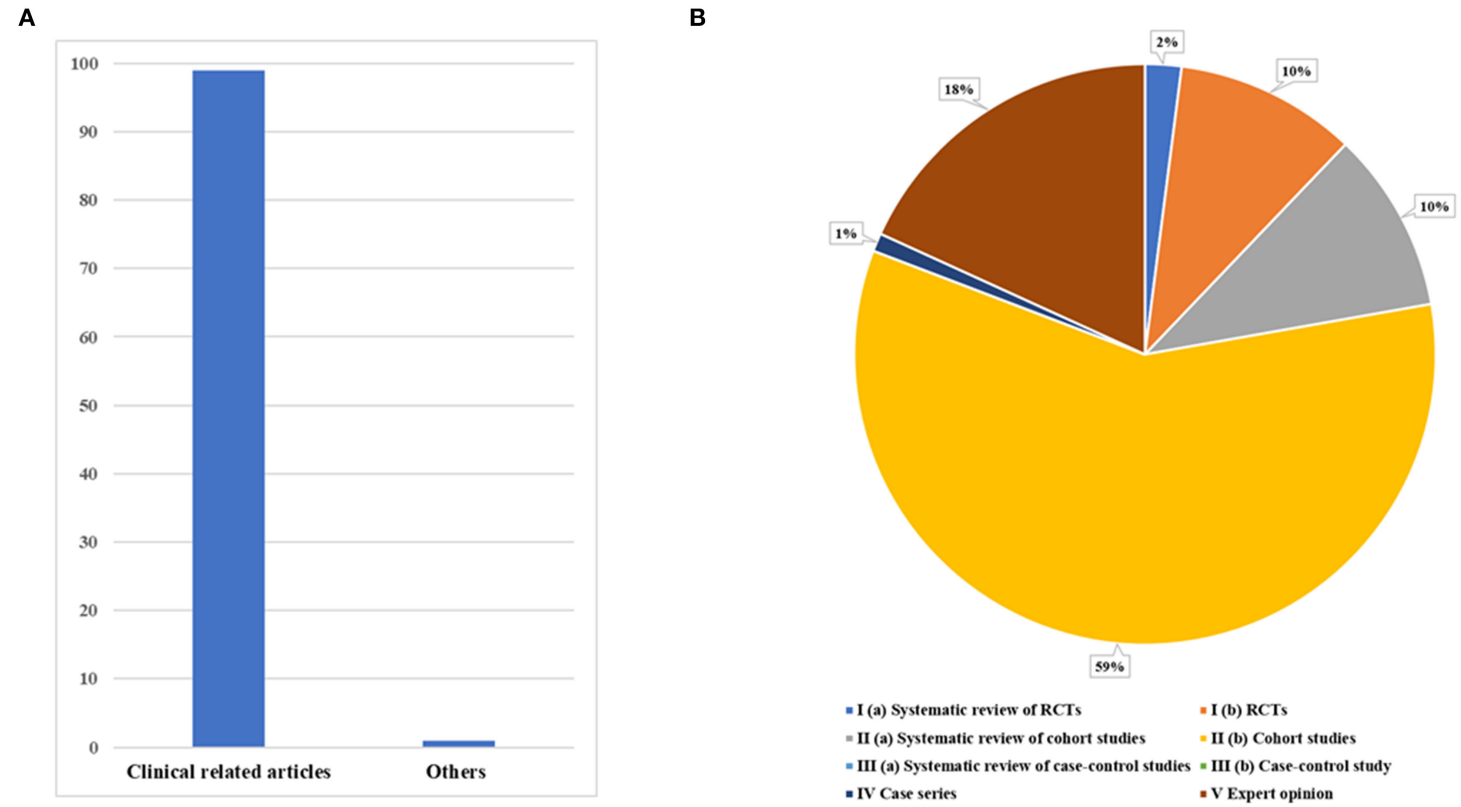
Study types of the 100 most-cited articles in the field of delirium. **(A)** The article classification. **(B)** The level of evidence for clinically relevant articles.

There were no animal or cell experiments in these articles. There were 99 clinical-related articles, and only one was about the delirium hypothetical model (Figure 3A). Since most of the articles were clinical-related articles, we further classified the clinical evidence levels of this kind of study (Figure 3B). In these articles, more than half of the highly cited articles were cohort studies with clinical evidence level II (b) (*n* = 58). Randomized controlled trials (RCTs) with clinical evidence grade I (b) accounted for 10% (*n* = 10), and expert opinions, systematic reviews, meta-analysis, and other summary studies accounted for 30% (*n* = 30).

### Keywords

**Figure 4:**
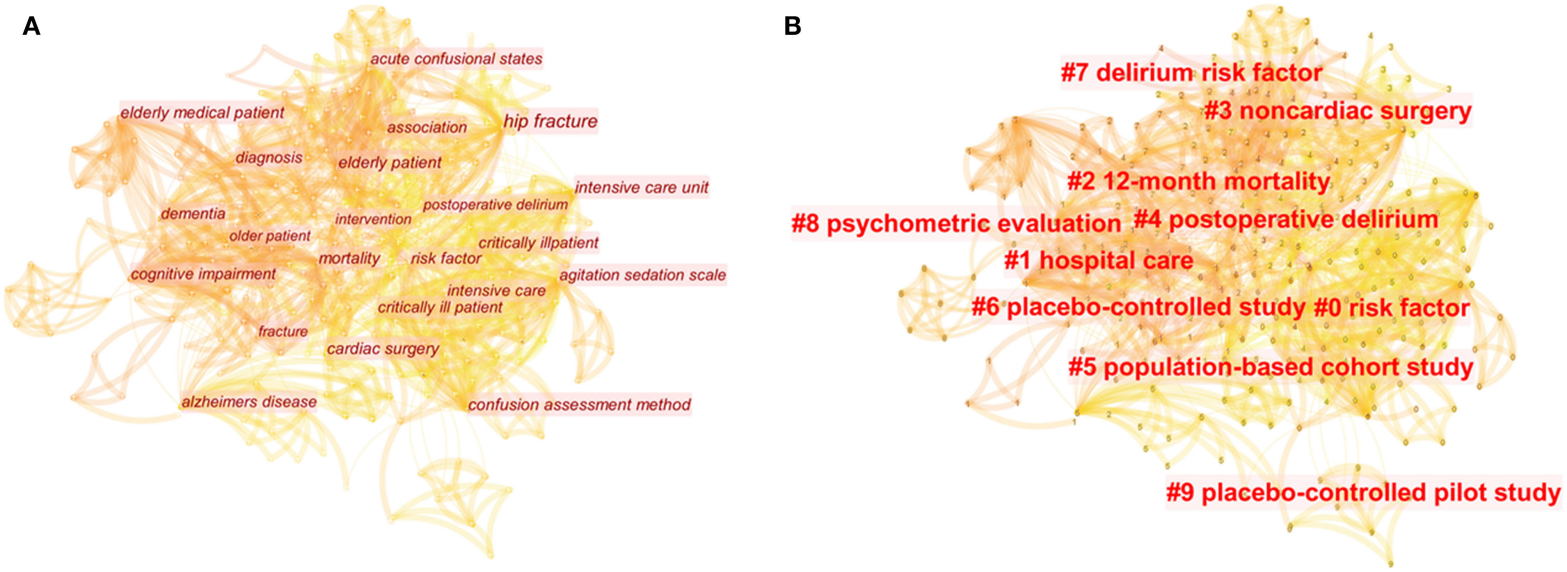
Keyword visualization analysis of the 100 most-cited articles in the field of delirium. **(A)** The map of keywords co-occurrence analysis. **(B)** The map of keywords cluster analysis.

We conducted a visual analysis of keywords in the top-100 articles by CiteSpace (Figure 4). Both keywords co-occurrence analysis and cluster analysis can be displayed in visual maps. Each node represents a keyword, and the node size represents the frequency of the keyword. That is to say, the larger the node is, the more frequently the keyword appears, which can indicate that the keyword may be the focus or hotspot of this research field. Besides, there will be a line connecting two nodes if they appear in one article at the same time, which means the two keywords have a co-occurrence relationship. The thicker the connection line is, the higher the co-occurrence frequency of the two keywords, which indicates that there may be a close relationship between them (16).

In total, the keyword co-occurrence analysis generated 298 nodes and 1,367 links (Figure 4A). The top-10 keywords with the highest frequency of co-occurrence were “confusion assessment method,” “risk factor,” “intensive care unit,” “mechanically ventilated patient,” “critically ill patient,” “elderly patient,” “dementia,” “acute confusional states,” “postoperative delirium,” and “hip fracture” (Table 4).

**Table 4:** The top-10 keywords with the highest frequency of co-occurrence.

**Keywords**	**Counts**
Confusion assessment method	24
Risk factor	22
Intensive care unit	21
Mechanically ventilated patient	19
Critically ill patient	18
Elderly patient	15
Dementia	15
Acute confusional states	12
Postoperative delirium	12
Hip fracture	12

Cluster analysis divided these keywords into 10 clusters, namely “risk factor” (#0), “hospital care” (#1), “12-month mortality” (#2), “non-cardiac surgery” (#3), and “postoperative delirium” (#4), “population-based cohort study” (#5), “placebo-controlled study” (#6), “delirium risk factor” (#7), “psychometric evaluation” (#8), and “placebo-controlled pilot study” (#9) (Figure 4B). The value of Q and Silhouette in CiteSpace are often used to determine whether clustering is reliable. In general, the Q value is >0.3 and the silhouette value is >0.7, which indicates that the clustering is reliable and worthy of attention. Our cluster analysis results show that the value of Q was 0.5827, and the silhouette values of all clusters were >0.70 (Table 5).

**Table 5:** The 10 clusters in the map of keyword co-occurrence.

**Cluster**	**Size**	**Silhouette**
Risk factor (#0)	62	0.875
Hospital care (#1)	46	0.753
12-month mortality (#2)	44	0.734
Non-cardiac surgery (#3)	34	0.909
Postoperative delirium (#4)	32	0.84
Population-based cohort study (#5)	31	0.922
Placebo-controlled study (#6)	16	0.927
Delirium risk factor (#7)	13	0.901
Psychometric evaluation (#8)	10	1
Placebo-controlled pilot study (#9)	8	0.988

## Discussion

The total number of citations for a published article indicates the importance of the published article in this practical field, and the analysis of the most influential publications is helpful to identify research hotspots in a particular field (9). In this study, we identified the 100 most-cited articles in the field of delirium and analyzed their related bibliometric indicators, study types, and keywords.

The results of publications and citations suggest that the overall publication of articles tends to be stable, and the number of citations is increasing year by year, indicating that the attention of this field continues to rise. The most-cited article is “*Clarifying confusion: the confusion assessment method. A new method for detection of delirium*” published by Inouye SK et al. in 1990 (17). They developed and validated a new standardized disorder assessment method (CAM) that allows non-psychiatric clinicians to quickly detect delirium in high-risk environments. Even today, CAM or its improved version is still used clinically, which is of great historical significance (18). It is worth mentioning that more than 1/5 of the 100 most-cited articles have his contribution, which means that Inouye SK is the most influential author in the field.

In addition, these articles were published between 1955 and 2018, while the total citation frequency of articles published in the last 3 years did not enter the top-100. This suggests that recently published papers need a period of exposure time to increase citation. Therefore, we introduce the definition of citation density so as not to miss the article which is published later but of great significance (19). We found that the article with the highest citation density is “*Clinical practice guidelines for the management of pain, agitation, and delirium in adult patients in the intensive care unit*” published by Barr Juliana et al. in 2013. This article provides an effective guide for the prevention and treatment of delirium in critically- ill patients (20).

We analyzed the countries/regions and institutions' distribution of these publications and found that the United States had the largest number of publications, followed by Canada. The total number of articles published in these two countries is more than 90% of the top-100 articles. Harvard University, Yale University, and Vanderbilt University in the United States are the top three institutions that publish the most top articles, accounting for more than half of the most-cited articles. This suggests that the United States is still at the forefront of the world in the field of delirium.

Journal analysis enables researchers to understand the journal distribution of the articles, which is helpful for researchers to choose journals in the field of delirium and to evaluate the overall quality of articles. Most articles have been published in *Critical Care Medicine*, and some articles have been published in top journals, such as *JAMA-Journal of the American Medical Association* and *New England Journal of Medicine*. And journals with high IF and in high JCR areas usually have high academic influence and attract more high-quality papers. We found that the journals that publish these articles are of high quality, almost all journals have high IFs, and most of the articles belong to the JCR Q1. These results indicate that the field of delirium is very mature and has attracted much attention from researchers.

In addition, most of these articles are clinical-related studies, which indirectly indicates that delirium is of high clinical concern at present, which may be related to its clinical incidence. In previous studies, review papers were usually cited the most frequently (21). However, our study found that cohort studies are the main cited articles in this field, and there are a large number of systematic reviews or meta-analyses of cohort studies. In order to evaluate the assessment of delirium and predict its risk factors, a cohort study is a more appropriate method, which we think is the reason for the large number of articles cited in this type of article (22, 23). There is only one non-clinically relevant article, which introduces a hypothetical model of delirium that suggests that drugs that restore microglial choline to control or directly inhibit neuroinflammation need clinical trials (24). Although this article can not be classified by the existing methods of classification of clinical evidence, it still provides ideas for clinical workers to guide the use of drugs and carry out therapeutic clinical trials.

Finally, we use visualization software to analyze the keywords of these articles, which is conducive to a further understanding of the various sub-directions involved. Keyword co-occurrence analysis can show the relationship between the main keywords, and cluster analysis is to classify keywords and group some keywords with the same attributes. Keyword co-occurrence analysis and cluster analysis of the top-100 articles can help readers sort out the hot areas of popular literature research and current research trends (8, 25). The results suggest that the research direction in the field of delirium focuses on the evaluation and treatment of delirium in critically ill patients and postoperative delirium (1, 26, 27). Furthermore, “confusion assessment method” and “risk factor” co-occur frequently, which is not surprising to us. Because if delirium can be effectively identified and prevented, the subsequent damage caused by the disease to patients can be reduced, and the key to effective prevention of delirium is to evaluate the risk factors (20). Cluster analysis is better to classify the related keywords into one category. Through cluster analysis, we find that risk factors for delirium, psychometric evaluation, hospital care, and various clinical study design are also hot topics in this field (28–30).

### Limitations

Our study has some limitations. The articles with the highest frequency of citation are those that have been published for a long time, which makes some newly published high-quality papers can not be cited enough to be analyzed by us (31). Furthermore, the keywords used in this study may not cover all the literature in this field, which is also an inherent limitation of bibliometrics (32). In addition, due to the time gap between the inclusion of the Web of Science and the publication of articles, some publications have not been included until 2022, and the citation times of articles are different when searching at different time points, so it is necessary to update them in future research.

## Conclusion

We summarized the 100 most-cited articles in the field of delirium to identify the current status and global trends. Our bibliometric analysis shows that the attention in this field continues to rise. The United States is still at the forefront of the world in this field. And researchers may prefer to cite the results of cohort studies. In addition, our results provide support for future research. Risk factors for delirium, psychometric evaluation, hospital care, and various clinical study design are still the focus of research.

## Data Availability Statement

The original contributions presented in the study are included in the article/Supplementary Material, further inquiries can be directed to the corresponding author.

## Ethics Statement

The study was exempted from institutional ethics review because only publicly available data were analyzed.

## Author Contributions

YG and FX: concept and design, administrative, technical, or material support. XF, QZ, and JW: acquisition, analysis, or interpretation of data. XF, QZ, and YG: critical revision of the manuscript for important intellectual content. XF and JW: statistical analysis. FX: supervision. All authors: drafting of the manuscript. All authors contributed to the article and approved the submitted version.

## Funding

This work was supported by the Special Scientific Research Project of Venous Thromboembolism Prevention (Heng Rui) of Sichuan Medical Association (Grant Number: 2019HR12), Special Support (Cultivation) Project Southwest Medical University (2022–2024), 2021 Sichuan Provincial Cadre Health Project (Grant Number: 2021-1505), and Cooperation Project of Luzhou Science and Technology Bureau and Southwest Medical University (Grant Number: 2019LZXNYDJ40).

## Conflict of Interest

The authors declare that the research was conducted in the absence of any commercial or financial relationships that could be construed as a potential conflict of interest.

## Publisher's Note

All claims expressed in this article are solely those of the authors and do not necessarily represent those of their affiliated organizations, or those of the publisher, the editors and the reviewers. Any product that may be evaluated in this article, or claim that may be made by its manufacturer, is not guaranteed or endorsed by the publisher.
