# Supplementary material for: Bibliometric Analysis of 100 Most-Cited Articles in Delirium
Source: Front Psychiatry. 2022 Jul 6;13:931632. doi: 10.3389/fpsyt.2022.931632 (PMC9298977; doi:10.3389/fpsyt.2022.931632)
Supplement: Supplementary file 1 [file Table_1.DOCX]

| **Supplementary Table 1** The 100 most-cited articles in the field of delirium | | | | | | |
| --- | --- | --- | --- | --- | --- | --- |
| **Rank** | **First Author** | **Title** | **Journal** | **Times Cited** | **Year** | **citation/year** |
| 1 | Inouye, SK | Clarifying confusion - the confusion assessment method - a new method for detection of delirium | *Annals of Internal Medicine* | 3091 | 1990 | 96.59 |
| 2 | Barr, J | Clinical Practice Guidelines for the Management of Pain, Agitation, and Delirium in Adult Patients in the Intensive Care Unit | *Critical Care Medicine* | 2008 | 2013 | 223.11 |
| 3 | Ely, EW | Delirium as a predictor of mortality in mechanically ventilated patients in the intensive care unit | *Jama-Journal of the American Medical Association* | 1748 | 2004 | 97.11 |
| 4 | Ely, EW | Delirium in mechanically ventilated patients - Validity and reliability of the Confusion Assessment Method for the intensive care unit (CAM-ICU) | *Jama-Journal of the American Medical Association* | 1684 | 2001 | 80.19 |
| 5 | Inouye, SK | A multicomponent intervention to prevent delirium in hospitalized older patients | *New England Journal of Medicine* | 1645 | 1999 | 71.52 |
| 6 | Inouye, SK | Delirium in elderly people | *Lancet* | 1477 | 2014 | 184.63 |
| 7 | Ely, EW | Evaluation of delirium in critically ill patients: Validation of the Confusion Assessment Method for the Intensive Care Unit (CAM-ICU) | *Critical Care Medicine* | 1229 | 2001 | 58.52 |
| 8 | Inouye, SK | Current concepts - Delirium in older persons | *New England Journal of Medicine* | 1159 | 2006 | 72.44 |
| 9 | Witlox, J | Delirium in Elderly Patients and the Risk of Postdischarge Mortality, Institutionalization, and Dementia A Meta-analysis | *Jama-Journal of the American Medical Association* | 1014 | 2010 | 84.50 |
| 10 | Anthony, JC | Limits of the mini-mental state as a screening-test for dementia and delirium among hospital patients | *Psychological Medicine* | 857 | 1982 | 21.43 |
| 11 | Inouye, SK | Precipitating factors for delirium in hospitalized elderly persons - Predictive model and interrelationship with baseline vulnerability | *Jama-Journal of the American Medical Association* | 838 | 1996 | 32.23 |
| 12 | Marcantonio, ER | Reducing delirium after hip fracture: A randomized trial | *Journal of the American Geriatrics Society* | 764 | 2001 | 36.38 |
| 13 | Bergeron, N | Intensive Care Delirium Screening Checklist: evaluation of a new screening tool | *Intensive Care Medicine* | 761 | 2001 | 36.24 |
| 14 | Pandharipande, P | Lorazepam is an independent risk factor for transitioning to delirium in intensive care unit patients | *Anesthesiology* | 717 | 2006 | 44.81 |
| 15 | Girard, TD | Delirium as a predictor of long-term cognitive impairment in survivors of critical illness | *Critical Care Medicine* | 692 | 2010 | 57.67 |
| 16 | Siddiqi, N | Occurrence and outcome of delirium in medical in-patients: a systematic literature review | *Age and Ageing* | 667 | 2006 | 41.69 |
| 17 | Ely, EW | the impact of delirium in the intensive care unit on hospital length of stay | *Intensive Care Medicine* | 646 | 2001 | 30.76 |
| 18 | Francis, J | A prospective-study of delirium in hospitalized elderly | *Jama-Journal of the American Medical Association* | 635 | 1990 | 19.84 |
| 19 | Marcantonio, ER | A clinical-prediction rule for delirium after elective noncardiac surgery | *Jama-Journal of the American Medical Association* | 609 | 1994 | 21.75 |
| 20 | Saczynski, JS | Cognitive Trajectories after Postoperative Delirium | *New England Journal of Medicine* | 597 | 2012 | 59.70 |
| 21 | Trzepacz, PT | Validation of the Delirium Rating Scale-revised-98: Comparison with the Delirium Rating Scale and the Cognitive Test for Delirium | *Journal of Neuropsychiatry and Clinical Neurosciences* | 591 | 2001 | 28.14 |
| 22 | Inouye, SK | A predictive model for delirium in hospitalized elderly medical patients based on admission characteristics | *Annals of Internal Medicine* | 577 | 1993 | 19.90 |
| 23 | Ouimet, S | Incidence, risk factors and consequences of ICU delirium | *Intensive Care Medicine* | 575 | 2007 | 38.33 |
| 24 | Leslie, DL | One-year health care costs associated with delirium in the elderly population | *Archives of Internal Medicine* | 539 | 2008 | 38.50 |
| 25 | Milbrandt, EB | Costs associated with delirium in mechanically ventilated patients | *Critical Care Medicine* | 532 | 2004 | 29.56 |
| 26 | Breitbart, W | the memorial delirium assessment scale | *Journal of Pain and Symptom Management* | 525 | 1997 | 21.00 |
| 27 | Devlin, JW | Clinical Practice Guidelines for the Prevention and Management of Pain, Agitation/Sedation, Delirium, Immobility, and Sleep Disruption in Adult Patients in the ICU | *Critical Care Medicine* | 523 | 2018 | 130.75 |
| 28 | Devlin, JW | Executive Summary: Clinical Practice Guidelines for the Prevention and Management of Pain, Agitation/Sedation, Delirium, Immobility, and Sleep Disruption in Adult Patients in the ICU | *Critical Care Medicine* | 506 | 2018 | 126.50 |
| 29 | Inouye, SK | Does delirium contribute to poor hospital outcomes? A three-site epidemiologic study | *Journal of General Internal Medicine* | 493 | 1998 | 20.54 |
| 30 | Inouye, SK | Nurses' recognition of delirium and its symptoms - Comparison of nurse and researcher ratings | *Archives of Internal Medicine* | 480 | 2001 | 22.86 |
| 31 | Pisani, MA | Days of Delirium Are Associated with 1-Year Mortality in an Older Intensive Care Unit Population | *American Journal of Respiratory and Critical Care Medicine* | 463 | 2009 | 35.62 |
| 32 | Lawlor, PG | Occurrence, causes, and outcome of delirium in patients with advanced cancer - A prospective study | *Archives of Internal Medicine* | 461 | 2000 | 20.95 |
| 33 | Trzepacz, PT | A symptom rating-scale for delirium | *Psychiatry Research* | 454 | 1988 | 13.35 |
| 34 | Fong, TG | Delirium in elderly adults: diagnosis, prevention and treatment | *Nature Reviews Neurology* | 449 | 2009 | 34.54 |
| 35 | Marcantonio, ER | Delirium is independently associated with poor functional recovery after hip fracture | *Journal of the American Geriatrics Society* | 446 | 2000 | 20.27 |
| 36 | McCusker, J | Delirium predicts 12-month mortality | *Archives of Internal Medicine* | 436 | 2002 | 21.80 |
| 37 | Isbell, H | An experimental study of the etiology of rum fits and delirium tremens | *Quarterly Journal of Studies On Alcohol* | 435 | 1955 | 6.49 |
| 38 | Morrison, RS | Relationship between pain and opioid analgesics on the development of delirium following hip fracture | *Journals of Gerontology Series A-Biological Sciences and Medical Sciences* | 428 | 2003 | 22.53 |
| 39 | Levkoff, SE | Delirium - the occurrence and persistence of symptoms among elderly hospitalized-patients | *Archives of Internal Medicine* | 410 | 1992 | 13.67 |
| 40 | Sikich, N | Development and psychometric evaluation of the pediatric anesthesia emergence delirium scale | *Anesthesiology* | 399 | 2004 | 22.17 |
| 41 | Salluh, JIF | Outcome of delirium in critically ill patients: systematic review and meta-analysis | *Bmj-British Medical Journal* | 398 | 2015 | 56.86 |
| 42 | Dubois, MJ | Delirium in an intensive care unit: a study of risk factors | *Intensive Care Medicine* | 395 | 2001 | 18.81 |
| 43 | Kalisvaart, KJ | Haloperidol prophylaxis for elderly hip-surgery patients at risk for delirium: A randomized placebo-controlled study | *Journal of the American Geriatrics Society* | 385 | 2005 | 22.65 |
| 44 | Fick, DM | Delirium superimposed on dementia: A systematic review | *Journal of the American Geriatrics Society* | 378 | 2002 | 18.90 |
| 45 | Inouye, SK | the dilemma of delirium - clinical and research controversies regarding diagnosis and evaluation of delirium in hospitalized elderly medical patients | *American Journal of Medicine* | 378 | 1994 | 13.50 |
| 46 | Breitbart, W | A double-blind trial of haloperidol, chlorpromazine, and lorazepam in the treatment of delirium in hospitalized AIDS patients | *American Journal of Psychiatry* | 376 | 1996 | 14.46 |
| 47 | Peterson, JF | Delirium and its motoric subtypes: A study of 614 critically ill patients | *Journal of the American Geriatrics Society* | 371 | 2006 | 23.19 |
| 48 | Chan, MTV | BIS-guided Anesthesia Decreases Postoperative Delirium and Cognitive Decline | *Journal of Neurosurgical Anesthesiology* | 367 | 2013 | 40.78 |
| 49 | Pandharipande, P | Prevalence and risk factors for development of delirium in surgical and trauma intensive care unit patients | *Journal of Trauma-Injury Infection and Critical Care* | 367 | 2008 | 26.21 |
| 50 | Thomason, JWW | Intensive care unit delirium is an independent predictor of longer hospital stay: a prospective analysis of 261 non-ventilated patients | *Critical Care* | 364 | 2005 | 21.41 |
| 51 | Aldecoa, C | European Society of Anaesthesiology evidence-based and consensus-based guideline on postoperative delirium | *European Journal of Anaesthesiology* | 355 | 2017 | 71.00 |
| 52 | Hshieh, TT | Effectiveness of Multicomponent Nonpharmacological Delirium Interventions A Meta-analysis | *Jama Internal Medicine* | 354 | 2015 | 50.57 |
| 53 | Marcantonio, ER | the relationship of postoperative delirium with psychoactive medications | *Jama-Journal of the American Medical Association* | 352 | 1994 | 12.57 |
| 54 | Robinson, TN | Postoperative Delirium in the Elderly Risk Factors and Outcomes | *Annals of Surgery* | 351 | 2009 | 27.00 |
| 55 | Lipowski, ZJ | Delirium in the elderly patient | *New England Journal of Medicine* | 347 | 1989 | 10.52 |
| 56 | Schor, JD | Risk-factors for delirium in hospitalized elderly | *Jama-Journal of the American Medical Association* | 341 | 1992 | 11.37 |
| 57 | Devlin, JW | Efficacy and safety of quetiapine in critically ill patients with delirium: A prospective, multicenter, randomized, double-blind, placebo-controlled pilot study | *Critical Care Medicine* | 334 | 2010 | 27.83 |
| 58 | Breitbart, W | the delirium experience: Delirium recall and delirium-related distress in hospitalized patients with cancer, their spouses/caregivers, and their nurses | *Psychosomatics* | 332 | 2002 | 16.60 |
| 59 | McNicoll, L | Delirium in the intensive care unit: Occurrence and clinical course in older patients | *Journal of the American Geriatrics Society* | 325 | 2003 | 17.11 |
| 60 | Su, X | Dexmedetomidine for prevention of delirium in elderly patients after non-cardiac surgery: a randomised, double-blind, placebo-controlled trial | *Lancet* | 323 | 2016 | 53.83 |
| 61 | Kain, ZN | Preoperative anxiety and emergence delirium and postoperative maladaptive behaviors | *Anesthesia and Analgesia* | 321 | 2004 | 17.83 |
| 62 | van Gool, WA | Systemic infection and delirium: when cytokines and acetylcholine collide | *Lancet* | 320 | 2010 | 26.67 |
| 63 | Lin, SM | the impact of delirium on the survival of mechanically ventilated patients | *Critical Care Medicine* | 320 | 2004 | 17.78 |
| 64 | Elie, M | Delirium risk factors in elderly hospitalized patients | *Journal of General Internal Medicine* | 319 | 1998 | 13.29 |
| 65 | OKeeffe, S | the prognostic significance of delirium in older hospital patients | *Journal of the American Geriatrics Society* | 314 | 1997 | 12.56 |
| 66 | Gaudreau, JD | Fast, systematic, and continuous delirium assessment in hospitalized patients: the Nursing Delirium Screening Scale | *Journal of Pain and Symptom Management* | 309 | 2005 | 18.18 |
| 67 | Pompei, P | Delirium in hospitalized older persons - outcomes and predictors | *Journal of the American Geriatrics Society* | 308 | 1994 | 11.00 |
| 68 | Bellelli, G | Validation of the 4AT, a new instrument for rapid delirium screening: a study in 234 hospitalised older people | *Age and Ageing* | 303 | 2014 | 37.88 |
| 69 | Girard, TD | Delirium in the intensive care unit | *Critical Care* | 300 | 2008 | 21.43 |
| 70 | Marcantonio, ER | Delirium in Hospitalized Older Adults | *New England Journal of Medicine* | 296 | 2017 | 59.20 |
| 71 | Rockwood, K | the risk of dementia and death after delirium | *Age and Ageing* | 296 | 1999 | 12.87 |
| 72 | Davis, DHJ | Delirium is a strong risk factor for dementia in the oldest-old: a population-based cohort study | *Brain* | 294 | 2012 | 29.40 |
| 73 | McCusker, J | Delirium in older medical inpatients and subsequent cognitive and functional status: a prospective study | *Canadian Medical Association Journal* | 293 | 2001 | 13.95 |
| 74 | Maldonado, JR | Neuropathogenesis of Delirium: Review of Current Etiologic theories and Common Pathways | *American Journal of Geriatric Psychiatry* | 291 | 2013 | 32.33 |
| 75 | Lipowski, ZJ | Transient cognitive disorders (delirium, acute confusional states) in the elderly | *American Journal of Psychiatry* | 290 | 1983 | 7.44 |
| 76 | Lynch, EP | the impact of postoperative pain on the development of postoperative delirium | *Anesthesia and Analgesia* | 289 | 1998 | 12.04 |
| 77 | Shehabi, Y | Delirium duration and mortality in lightly sedated, mechanically ventilated intensive care patients | *Critical Care Medicine* | 288 | 2010 | 24.00 |
| 78 | Radtke, FM | Monitoring depth of anaesthesia in a randomized trial decreases the rate of postoperative delirium but not postoperative cognitive dysfunction | *British Journal of Anaesthesia* | 287 | 2013 | 31.89 |
| 79 | Rudolph, JL | Derivation and Validation of a Preoperative Prediction Rule for Delirium After Cardiac Surgery | *Circulation* | 285 | 2009 | 21.92 |
| 80 | Inouye, SK | A chart-based method for identification of delirium: Validation compared with interviewer ratings using the confusion assessment method | *Journal of the American Geriatrics Society* | 284 | 2005 | 16.71 |
| 81 | Dyer, CB | Postoperative delirium - a review of 80 primary data-collection studies | *Archives of Internal Medicine* | 284 | 1995 | 10.52 |
| 82 | Han, L | Use of medications with anticholinergic effect predicts clinical severity of delirium symptoms in older medical inpatients | *Archives of Internal Medicine* | 282 | 2001 | 13.43 |
| 83 | Reade, MC | Sedation and Delirium in the Intensive Care Unit | *New England Journal of Medicine* | 281 | 2014 | 35.13 |
| 84 | Hshieh, TT | Cholinergic deficiency hypothesis in delirium: A synthesis of current evidence | *Journals of Gerontology Series A-Biological Sciences and Medical Sciences* | 281 | 2008 | 20.07 |
| 85 | Oh, ES | Delirium in Older Persons Advances in Diagnosis and Treatment | *Jama-Journal of the American Medical Association* | 279 | 2017 | 55.80 |
| 86 | Fong, TG | Delirium accelerates cognitive decline in Alzheimer disease | *Neurology* | 279 | 2009 | 21.46 |
| 87 | Girard, TD | Feasibility, efficacy, and safety of antipsychotics for intensive care unit delirium: the MIND randomized, placebo-controlled trial | *Critical Care Medicine* | 273 | 2010 | 22.75 |
| 88 | Dasgupta, M | Preoperative risk assessment for delirium after noncardiac surgery: A systematic review | *Journal of the American Geriatrics Society* | 273 | 2006 | 17.06 |
| 89 | Rudolph, JL | Postoperative Delirium: Acute Change with Long-Term Implications | *Anesthesia and Analgesia* | 272 | 2011 | 24.73 |
| 90 | Skrobik, YK | Olanzapine vs haloperidol: treating delirium in a critical care setting | *Intensive Care Medicine* | 272 | 2004 | 15.11 |
| 91 | Jackson, JC | the association between delirium and cognitive decline: A review of the empirical literature | *Neuropsychology Review* | 268 | 2004 | 14.89 |
| 92 | Zaal, IJ | A Systematic Review of Risk Factors for Delirium in the ICU | *Critical Care Medicine* | 266 | 2015 | 38.00 |
| 93 | Inouye, SK | Delirium: A symptom of how hospital care is failing older persons and a window to improve quality of hospital care | *American Journal of Medicine* | 265 | 1999 | 11.52 |
| 94 | Balas, MC | Effectiveness and Safety of the Awakening and Breathing Coordination, Delirium Monitoring/Management, and Early Exercise/Mobility Bundle | *Critical Care Medicine* | 264 | 2014 | 33.00 |
| 95 | Lipowski, ZJ | Delirium (acute confusional states) | *Jama-Journal of the American Medical Association* | 264 | 1987 | 7.54 |
| 96 | Maldonado, JR | Dexmedetomidine and the Reduction of Postoperative Delirium after Cardiac Surgery | *Psychosomatics* | 261 | 2009 | 20.08 |
| 97 | Francis, J | Prognosis after hospital discharge of older medical patients with delirium | *Journal of the American Geriatrics Society* | 257 | 1992 | 8.57 |
| 98 | van Rompaey, B | Risk factors for delirium in intensive care patients: a prospective cohort study | *Critical Care* | 255 | 2009 | 19.62 |
| 99 | Marcantonio, ER | the association of intraoperative factors with the dcevelopment of postoperative delirium | *American Journal of Medicine* | 255 | 1998 | 10.63 |
| 100 | Clegg, A | Which medications to avoid in people at risk of delirium: a systematic review | *Age and Ageing* | 251 | 2011 | 22.82 |
